# Development of a low-fructose carbohydrate gel for exercise application

**DOI:** 10.1016/j.heliyon.2024.e33497

**Published:** 2024-06-22

**Authors:** Isabel G. Martinez, Michael J. Houghton, Matteo Forte, Gary Williamson, Jessica R. Biesiekierski, Ricardo J.S. Costa

**Affiliations:** a Department of Nutrition, Dietetics and Food, Monash University, Level 1, 264 Ferntree Gully Road, Notting Hill, VIC 3168, Australia; b Victorian Heart Institute, Monash University, Victorian Heart Hospital, 631 Blackburn Road, Clayton, VIC 3168, Australia; c Department of Land, Environment, Agriculture and Forestry, Università Degli Studi di Padova, Viale Dell’Università 16, 35020, Legnaro, PD, Italy

**Keywords:** Endurance running, Glucose availability, Carbohydrate malabsorption, Feeding tolerance, Gastrointestinal symptoms

## Abstract

This study aimed to develop a low-fructose (<3 g/serve) carbohydrate (CHO) gel for athletes. Various prototypes with 30 g CHO/serve and differing water content (12 %, 21 %, 32 %, 39 % w/v) were created and evaluated for sensory attributes. The final gel contained 62.1 ± 0.2 g CHO/100 g with 0.17 % *w/w* fructose. Endurance athletes (n = 20) underwent a feeding-challenge protocol, ingesting 30 g gel every 20 min during 2 h of running (60 % $\dot{V}$O_2_max), followed by a 1 h self-paced distance test. Blood glucose increased significantly from baseline (4.0 ± 0.9 vs. 6.6 ± 0.6 mmol/L, *p* < 0.001) and remained elevated after the distance test (4.9 ± 0.7 mmol/L, *p* < 0.05). Breath hydrogen levels increased (5 ± 4 ppm, *p* < 0.05) without substantial CHO malabsorption detected. Gastrointestinal symptoms (GIS) increased during exercise but were mild. The low-fructose CHO gel demonstrated good tolerance, promoting glucose availability without severe GIS or CHO malabsorption.

## Highlights

• Carbohydrate gels are a convenient fuelling option during exercise.
• Commercially-available gels with both glucose and fructose may not be well-tolerated by everyone.
• Quantification of sugars confirmed the actual content of the developed low-fructose gel.
• Assessment of blood glucose, malabsorption, and gut symptoms offer insights on individual tolerance.
• The developed low-fructose gel promotes fuel availability and was well-tolerated during running.

## 1 Introduction

Consuming carbohydrates (CHO) during prolonged exercise modulates blood glucose levels for skeletal muscle use [1]. CHO taste-receptors in the mouth also activate the central nervous system, enhancing endurance performance [2]. Compared to whole foods, CHO supplements (i.e., gels, chews, or liquid solutions) are higher in CHO, with some absorbed faster after ingestion [3]. CHO gels and liquid solutions are oxidized at similar rates during exercise [4], but a greater volume of CHO liquid solution is required to meet target intakes, without individual factors considered (e.g., fluid loss rate, environmental temperature and humidity). This may negatively impact an individual's appetite, feeding tolerance, incidence and severity of gastrointestinal symptoms (GIS), and hydration status during exercises [5,6]. As such, from a practical perspective, CHO gels are considered as a convenient and well-tolerated option [7].

Fuelling using multiple transportable CHOs during prolonged exercise (>2.5–3 h) has been proposed to enhance CHO absorption and availability for skeletal muscle oxidation [8]. Previous sports nutrition guidelines recommended up to 90 g CHO/h for activities lasting > 3h, preferably from a mix of glucose and fructose [9]. However, previous studies have demonstrated poor tolerance to load (90 g) and CHO type (e.g., multiple transportable CHOs) [3,10]. Updated guidelines now suggest a broader intake range (30–90 g CHO/h), considering individual needs, tolerance, practicality, and CHO blends [1,5,6].

Certain types of short-chain CHOs known as fermentable oligosaccharides, disaccharides, monosaccharides, and polyols (FODMAP) cannot be fully digested or are incompletely absorbed in the small intestine and are known to induce GIS in some individuals [11]. FODMAPs are naturally present in many foods such as lactose in dairy products, sorbitol and mannitol in sweetened food, and fructose in fruits, honey, and certain sports food. Insights on fructose malabsorption and its impact on gastrointestinal physiology [12,13], along with observed GIS when consuming high fructose sports products [3,10,14], highlights the need to vary CHO blends, possibly reducing the presence of this sugar. This may also be a consideration for individuals following a diet low in FODMAP to manage GIS, within both exercise and clinical settings [11,15].

Commercially-available CHO supplements for exercise come in varying formulations, flavours, and forms. Adding to the complexity are marketing claims on ideal consistency and sugar ratios to improve fuel availability and reduce GIS risk, often lacking scientific evidence [16]. Athletes and supporting practitioners may then resort to experimenting with creating a tailored formulation using commercially-available ingredients, yet there is currently no guidance on the development process to ensure product quality and safety. To address this gap, the current applied experimental design aims to test a developmental procedure encompassing market sourcing, product formulation, physicochemical, microbiological and sensoryl analysis, and investigate tolerance to a low-fructose CHO gel for exercise.

## 2 Methods

Ethical approval for the involvement of human subjects in this study was granted by Monash University Human Research Ethics Committee (MUHREC), reference number 35216, August 19, 2022. Participants provided written consent to participate in the study.

### 2.1 Product development and testing

**Fig. 1:**
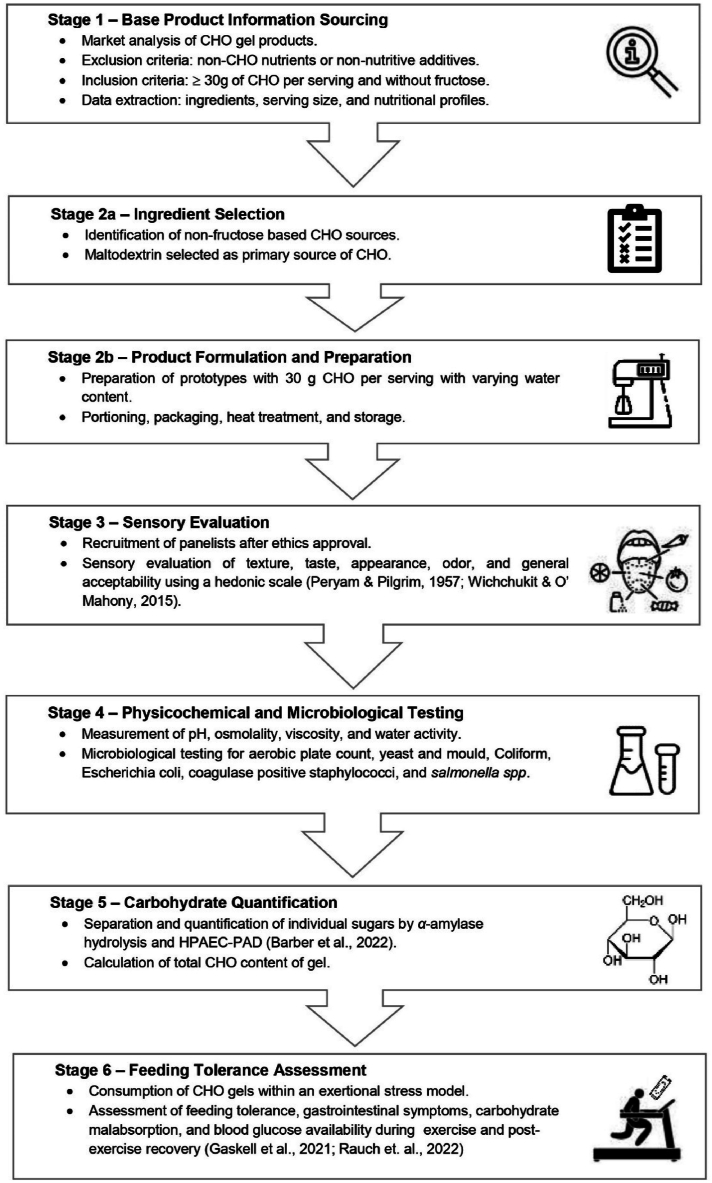
Overview of processes undertaken for product development and testing.

A six-stage method was followed, starting with gathering product information and concluding with a feeding challenge. Fig. 1 summarises these stages, which are further detailed below.

#### 2.1.1 Base product information sourcing

A survey of CHO gels available in the local commercial market (i.e., supermarkets, health stores, and online sports supplement retailers) in Australia was conducted. Products containing other nutrients (e.g., medium chain triglyceride oil or amino acids) or non-nutritive additives (e.g., caffeine) were excluded. Products with at least 30 g CHO/packet and/or meeting 30 g CHO/h intake based on serving suggestion and with no fructose listed in the ingredients were included as benchmark products, and ingredients, serving size, and nutritional profile were extracted (Table 1).

**Table 1:** Nutrition information of benchmark CHO gel products (n = 11).

Product	Serving Size (g per gel)	Recommended intake^a^ (gel per h)	Nutrition per serving							Ingredients
			Energy (kcal)	Total CHO (g)	Sugars (g)	Fat (g)	Protein (g)	Fiber (g)	odium (mg)	
**A**	27	1–2	84	21	13	0	0.5	NA	59	Rice syrup, natural flavoring, salt, preservatives, and flavor enhancer.
**B**	31	1–2	100	23	17	0	1	2	100	Strawberry, raw coconut palm nectar, blackstrap molasses, and pink himalayan salt.
**C**	34	1–2	106	22	12	0	0	1	58	Maltodextrin, cane sugar, water, raspberry juice concentrate, sea salt, potassium citrate, and citric acid.
**D**	35	1–2	86	21	4.2	0	0.2	0.2	54	Maltodextrin, filtered water, organic cane sugar, lemon juice (4 %), lime juice (3 %), orange juice (1 %), mineral salt (sodium citrate), sea salt, preservative (potassium sorbate, sodium benzoate), and antioxidant (ascorbic acid).
**E**	40	1–3	91	23	3	0	0	NA	50	Water, glucose syrup, maltodextrin, fruit juice concentrate (raspberry 1 %, blackcurrant, blueberry 0.2 %), acidity regulators (sodium citrates, citric acid, malic acid), sodium chloride, preservatives (potassium sorbate, sodium benzoate), and natural flavorings.
**F**	42	1–2	100	21	14	0.5	1	2	105	Blueberry puree, cane sugar, water, brown rice syrup, powdered chia seeds, blueberry concentrate, sea salt, and citric acid.
**G**	45	1–2	117	30	2.9	0	0	NA	36	Maltodextrin, filtered water, acidity regulator (citric acid), natural flavor, mineral salts (potassium chloride, salt), and preservative (sodium benzoate).
**H**	45	*Not specified*	118	30	2.9	0	0	NA	36	Maltodextrin, filtered water, citric acid (330), flavor, potassium chloride, salt, and sodium benzoate (211).
**I**	54	1–2	180	45	22	0	2	2	85	Basmati rice, apple juice, organic apple sauce, yams, maple syrup, organic lemon juice, sea salt, cinnamon, and vanilla.
**J**	60	1–3	87	22	0.6	0	0	0	10	Water, maltodextrin (from maize) gelling agents (gellan gum, xanthan gum), natural flavoring, acidity regulators (citric acid, sodium citrate), preservatives (sodium benzoate, potassium sorbate), sweetener (acesulfame K), sodium chloride, and antioxidant (ascorbic acid).
**K**	76	1–2	200	50	23	0	0	0	100	Water, maltodextrin (maltodextrin DE15, highly branched cyclic dextrin (Cluster Dextrin®), maltodextrin DE6), corn glucose syrup, sucrose, natural flavor, acidity regulators (citric acid, sodium citrate), sodium chloride, gelling agent (gellan gum), and preservative (potassium sorbate).

CHO = carbohydrate.

a Recommended intake based on manufacturer's instruction as presented on the product label.

##### 2.1.1.1 Ingredient selection

Ingredients were chosen based on available product information, considering readily-available options with minimal fructose (<2.5 g/100 mL liquid food or < 5.0 g/100 g solid food) [17,18]. The selected ingredients were maltodextrin (Malto-Dextrin DE 18–20, Bioflex Nutrition Pty. Ltd., Crabtree, Tasmania, Australia) and glucose syrup (Queens Glucose Syrup, Dr. Oetker Pty. Ltd., Alderley, Queensland, Australia). The solution was diluted with water, and flavoured using a sucrose-based syrup (Cottee's Chocolate Flavoured Syrup, Kraft Heinz Australia Pty. Ltd., Southbank, Victoria, Australia).

##### 2.1.1.2 Product formulation and preparation

The target CHO content of the product was 30 g/per serve with a fructose content less than 3 g per serving [18]. Ingredient ratios were calculated to ensure that maltodextrin and glucose syrup constituted the majority of CHOs (86 %). Prototypes with varying water content, mirroring the average serving size of commercially-available gels, were created and coded as Sample A (12 % *w/v*), B (20 % *w/v*), C (32 % *w/v*), and D (39 % *w/v*). All ingredients were combined, dispersed in water, and mixed for 10 min using an immersion blender (Bosch ErgoMixx MSM66120, Robert Bosch Pty Ltd, Clayton, Victoria, Australia). The product was packaged in a foil pouch (Spout Pouches 10 mm, Pouch Direct Pty. Ltd., Smithfield. New South Wales, Australia). A ‘*kill step’* involved submerging the filled pouch in a boiling water bath for 5 min until the mixture reached 100 °C, with periodic agitation. The packaged product was cooled to room temperature before storage. Production took place in the commercial kitchen of BASE Facility (Monash University, Notting Hill, Victoria, Australia).

#### 2.1.2 Sensory evaluation

Samples A, B, C, and D underwent sensory evaluation with 10 volunteers (age: 39 ± 11 years, sex: 6 females and 4 males) to identify the preferred formulation and assess acceptability. Ethnicity of panellists included both Caucasians (n = 7) and Asians (n = 3). Six out of the 10 volunteers were endurance athletes who have previous experience of using CHO gels during exercise. The sensory evaluation was performed under standardised conditions, wherein all samples were prepared and presented at the same temperature in neutral vessels randomly coded for anonymity. The order of sample presentation was randomised and panellists were provided with water and were instructed to consume it in between tasting samples. Using a 9-point hedonic scale (9 – like very much, 1- dislike very much) [19,20], the samples were assessed for the following attributes: texture, taste, colour, odour, and general acceptability, including suitability for use during exercise. The mean values of acceptability for each product were then calculated and analysed using appropriate statistical tests.

#### 2.1.3 Physicochemical and microbiological testing

Osmolality was measured in triplicate (coefficient of variation, CV: 0.9 %) using a freeze-point osmometer (Osmomat 030, Gonotec, Berlin, Germany). Viscosity, in millipascal-second (mPa·s), was determined using a viscometer (DV-I Prime, Brookfield Engineering Laboratories, U.S.A) with spindle number 64, in triplicate and at room temperature (CV: 0.6 %). The pH (pH Test Strips, SIGMA Chemical Company, St. Louis, Missouri, USA) of the product was also measured in triplicate. Water activity (a_w_) and microbiological testing (SGS Australia Pty Ltd., Notting Hill, Victoria, Australia) were conducted, covering aerobic plate count, yeast and mould, coliform, *Escherichia coli*, coagulase positive *Staphylococcus*, and *Salmonella* spp. tests. Subsequent aerobic plate count and yeast and mould tests were (day 29 and 126 post-production) performed.

#### 2.1.4 CHO quantification

Monosaccharides, disaccharides, and maltooligosaccharides in the CHO gel were separated and quantified by high-performance anion-exchange chromatography with pulsed amperometric detection (HPAEC-PAD), using our published protocol [21]. The gel was digested *in vitro* with human salivary *α-*amylase (EC 3.2.1.1) to hydrolyse longer maltooligosaccharide molecules (>5 glucose monomers), not detected by HPAEC-PAD, into measurable maltooligosaccharides, maltose, and glucose to calculate total CHO.

The analysis was conducted in triplicate using ultrapure water (18.2 MΩ•cm at 25 °C) from a MilliQ system (Merck Life Science, Bayswater, VIC, Australia). The gel was warmed to 37 °C and diluted in warm water to 83.3 g gel/L to decrease its viscosity. For *in vitro* digestion, aliquots were mixed 1/1 (v/v) with freshly prepared human salivary *α*-amylase IX (Merck Life Science) reconstituted and diluted in pre-warmed phosphate buffered saline (PBS) to provide a final assay concentration of 0.70 U. Controls were mixed with PBS only. The addition of *α*-amylase initiated the digestion, with tubes immediately incubated in a 37 °C water bath with gentle agitation for up to 240 min. After incubation, tubes were placed onto dry ice to stop the reaction and stored at −80 °C, so all samples could be prepared and analysed for HPAEC-PAD together. Separation of mono- and disaccharides and maltooligosaccharides were achieved on the Dionex CarboPac PA210-Fast-4μm and CarboPac SA10-4 μm analytical columns, respectively [21].

Duplicate injections were performed and peak identification was achieved by comparing retention times to sugar standards. Chromatograms were processed using Dionex Chromeleon 7 Chromatography Data System software, ensuring peaks were suitably integrated before recording peak area. Sugars were quantified from peak areas using standard curves. For mono-/disaccharides, standards included glucose, sucrose, fructose, and maltose (Merck Life Science). Additionally, standards of erythritol, myo-inositol, l-rhamnose, galactose, isomaltose, lactose, and lactulose are routinely ran to detect presence of these sugars. For maltooligosaccharides, the standard mixture comprised of maltose, maltotriose (Mal-3), maltotetraose (Mal-4), and maltopentaose (Mal-5) (Megazyme, Bray, Ireland). Standards were analysed using the same protocol. Data were processed in Excel (Microsoft, Redmond, WA, USA), calculating individual sugar and total CHO content per 100 g of the gel. Chromatograms and standard curves were drawn using GraphPad Prism 9 (GraphPad Software, Boston, MA, USA).

#### 2.1.5 Feeding tolerance assessment

**Fig. 2:**
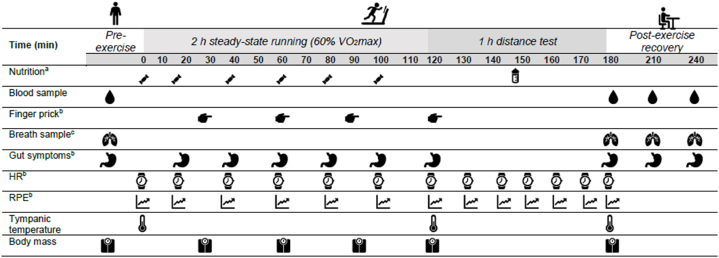
Gut-challenge protocol for feeding tolerance assessment of CHO gel. ^a^ One CHO gel with 290 mL water during the first 2 h steady-state running and *ad libitum* water during the 1 h distance test. ^*b*^ Collected prior to provision of nutrition. ^c^ Breath sample and gut symptoms collected every 15 min during the post-exercise recovery period.

The developed product was used in a modified gut challenge protocol [3,10] to assess feeding tolerance during exercise with running as the exercise modality (Fig. 2) [22].

Twenty recreationally competitive endurance athletes (age: 35 ± 10 years, weight: 74.2 ± 12.9 kg, height: 1.73 ± 0.08 cm, fat-free mass: 56.18 ± 12.4 kg, $\dot{V}$*O*_2max_: 52.2 ± 7.4 ml/kg/min, and % females: 20) participated in the feeding challenge assessment. Baseline height (stadiometer, Holtain Limited, Crosswell, Crymych, UK), body mass (BM), and composition (mBCA 515, Seca, Ecomed, Hamburg, Germany) were collected. Maximal oxygen uptake ($\dot{V}$O_2max_, Vyntus, Vyaire Medical, Macquarie Park New South Wales, Australia) was estimated through an incremental run test until volitional exhaustion on a motorised treadmill (Pulsar, h/p/cosmos, AI Medical International Pty. Ltd., North Adelaide, South Australia, Australia). The speed equivalent to 60 % $\dot{V}$O_2max_ at 1 % gradient was used as the running speed for the trials.

A high-CHO control diet (43 ± 10 kcal and 8 ± 2 g CHO per kg BM) was consumed the day before the exercise trial. A standardised low-fibre and FODMAP breakfast (553 ± 140 kcal, 70 ± 16 g CHO, 16 ± 5 g protein, 6 ± 3 g fat, 3 ± 1 g fibre, and 2 ± 1 g total FODMAP) with 400 mL of water was consumed 2.5 h prior to the start of the run. Whole blood samples were collected into a 6 mL lithium heparin vacutainer tube (Greiner Vacuette, Interpath, Somerton, Victoria, AU) from the antecubital vein via venepuncture immediately before and after exercise, and 1 h and 2 h post-exercise. Body temperature (Omron TH839S, Kyoto, Japan) and nude body mass (Seca 813, Ecomed Hamburg, Germany) were measured at pre- and post-exercise. Additionally, clothed body mass was monitored every 30 min during the first 2 h of the run. Breath samples were collected in a breath collection bag (Wagner Analysen Technick, Bremen, Germany) at pre- and post-exercise. GIS were assessed using the modified visual analogue scale (mVAS) assessment tool [23] before, during, and post-exercise. The developed CHO gel was ingested upon the start (0 min) and every 20 min thereafter (20-, 40-, 60-, 80-, and 100-min) during the first 2 h of exercise (90 g/h CHO) with 290 mL water, followed by a 1 h self-paced distance test with *ad libitum* water. The volume of water provided with the gel was standardised based on previous evidence showing that 10 % w/v concentration facilitates gastric emptying and reduces upper-GIS risk [24]. Heart rate (HR), rating of perceived exertion (RPE), and GIS were recorded every 20 min prior to provision of the CHO gel and water during the first 2 h of the run and every 10 min during the 1 h distance test. Capillary blood glucose was measured every 30 min during the first 2 h of the run (Accu-check Performa, Roche, North Ryde, New South Wales, Australia), in duplicate (CV: 0.1 %). All trials were conducted at 23.0 ± 1.0 °C ambient temperature (T_amb_) and 49.3 ± 6.3 % relative humidity (RH).

Haemoglobin (HemoCue Hb 201^+^, Ängelholm, Sweden) and blood glucose (HemoCue Glucose 201 RT, HemoCue AB, Ängelholm, Sweden) were measured in duplicate (CV: 0.1 % and 0.2 %, respectively). Haematocrit was measured in triplicate (CV: 0.1 %) using capillary tubes (Westlab, Mitchell Park, Victoria, Australia) with heparin whole blood, centrifuged at 9000 g for 2 min. Haemoglobin and haematocrit values were used to estimate changes in plasma volume relative to baseline [25]. The remaining heparin blood samples were centrifuged at 1500 g for 10 min at 4 °C and 50 μg heparin plasma was used to measure plasma osmolality (P_osmol_), in duplicate (CV: 0.1 %). The remaining plasma was aliquoted and stored at −80 °C. Hydrogen and methane content of breath samples were measured in duplicate (CV: 1.2 %) using a gas sensitive analyser (Breathtracker Digital Microlyzer, Quintron, Milwaukee, Wisconsin, US) [26].

### 2.2 Statistical analysis

Using mean, standard deviation (SD), and effect size data from prior studies with a similar protocol [3,10]; and applying a standard *α* (0.05) and *β* (0.08) values, a sample size of 19 was determined for sufficient statistical power (G*Power 3.1, Kiel, Germany). SPSS (IBM Corp. Released 2020. IBM SPSS Statistics for Windows, Version 29.0. Armonk, NY: IBM Corp) was used for statistical analysis. Skewness and kurtosis coefficients were calculated to determine appropriate statistical test. Paired-sample test or the non-parametric Wilcoxon signed-rank test was used to assess significant differences between variables. Analysis of variance (ANOVA), followed by Tukey test was used to determine significant differences in the sensory testing results. The significance level was set at *p* < 0.05. Mean ± SD is presented in figures and tables, unless specified otherwise. GIS and feeding tolerance variables are presented as an accumulative score or individual participant range.

## 3 Results

### 3.1 Base product information sourcing

Twenty-four CHO gels were identified in the local commercial market (Melbourne, Victoria, Australia), with 9 products containing other nutrients/additives excluded. Among these, only 12 gels contained at least 30 g CHO/serving or based on product serving suggestion and only 11 were free from fructose or sucrose based on product labels. Table 1 details the nutrition information of the benchmark products, with brand names kept confidential to comply with product anonymity ethical procedures.

Among the identified 11 benchmark products, the mean serving size was 47 ± 14 g per serve, providing an average of 2.7 ± 0.5 kcal/g of product. Maltodextrin was used as the primary source of CHOs in majority of the products (64 %). While some of the products did not have fructose listed on the ingredient list, these contained fruit-based ingredients (e.g., apple juice, blueberry puree, raspberry juice, or apple sauce) or other potentially high-FODMAP ingredients (e.g., coconut nectar, molasses). Overall, the nutritional content (range of CHO: 21–50 g/packet), and the recommended dosage and timing of intake (1–3 packets/h) varied greatly among the identified products (Table 1).

### 3.2 Product formulation and preparation

The developed gel is comprised of maltodextrin (38 %), glucose syrup (21 %), water (2 %), and chocolate-flavoured syrup (20 %). The sucrose-based syrup used for flavoring contains sodium metabisulphite, modified starch thickener, cocoa powder, chocolate flavour, citric acid, salt, and xanthan gum with undisclosed amounts on the label. Calculations based on ingredient labels indicate that a serving of the developed CHO gel (47 g) provides 123 kcal and 30 g of CHOs (Appendix A, Table S1).

### 3.3 Sensory evaluation

**Fig. 3:**
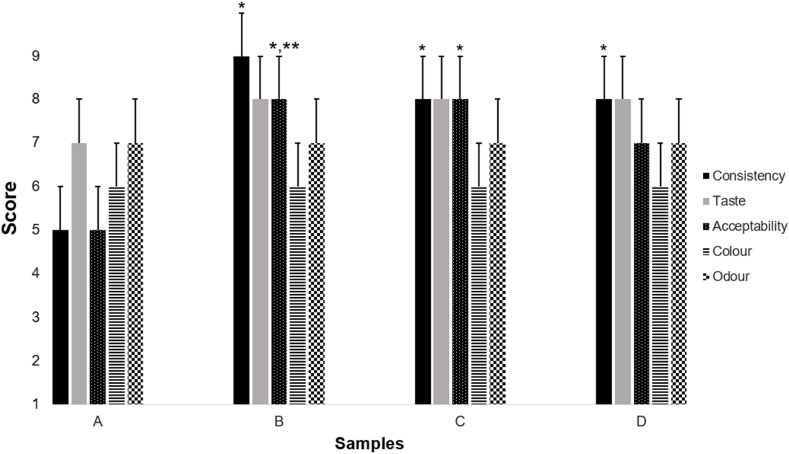
Sensory evaluation results of prototypes with varying water content: A (12 % w/v), B (21 % w/v), C (32 % w/v), and D (39 % w/v). Mean ± SD (n = 10). *p < 0.001 vs. sample A, **p < 0.001 vs. sample D.

The developed prototypes had good sensorial properties, with positive (ranging from 6 – like slightly to 9 – like very much) or neutral (5 – neither like nor dislike) scores for consistency, taste, colour, odour, and overall acceptability. For consistency, Sample A was least preferred versus the others (*p* < 0.05), with Sample B ranked highest but not significantly different from Samples C and D. For general acceptability, Sample B and C were ranked higher than Samples A and D (*p* < 0.05). All samples were similar for the rest of the attributes (Fig. 3). Considering these results and aiming to minimize serving size, Sample B was selected as the final formulation.

### 3.4 Physicochemical and microbiological testing

The developed product's mean osmolality and viscosity were 171 ± 1.5 mOsm and 5466 ± 34 mPa·s, respectively. The product had a pH of 4, water activity of 0.90 a_w_, and negligible counts for aerobic plate count, yeast and mould, Coliform, *Escherichia coli*, coagulase positive *Staphylococci*, and *Salmonella* spp (Appendix A*,* Table S2*)*.

### 3.5 CHO quantification by α-amylase hydrolysis and HPAEC-PAD

All sugars separated well, with distinct peaks for glucose, sucrose, fructose, and maltose on the PA210 column. No other sugars were detected and fructose was very low, close to the limit of detection. Spiking the samples with fructose standard confirmed trace amounts (0.17 % *w/w*) present. The maltooligosaccharides present in the samples eluted from the PA210 column with the subsequent 1–2 injections with no interference with the mono-/disaccharide peaks. Maltose, Mal-3, Mal-4, and Mal-5 eluted with good resolution from the SA10 column. The mean CV for maltose and all sugars were 0.4 % and 4.8 %, respectively. Mean maltose content was calculated for each sample from both analyses.

Hydrolysis of the gel was facilitated by incubation at 37 °C with human salivary α-amylase for ≤4 h. Glucose, maltose, Mal-3 and Mal-4 all increased during the digestion, while Mal-5 decreased and was absent, or too low to be detected, by 30 min. Sucrose or fructose content remained unchanged and isomaltose was either undetected or below the limit of detection post-hydrolysis. Glucose and maltose production indicated >90 % completion after 30 min, with no further products formed after 120 min. Total CHO was calculated based on sugar contents recorded after 120 min hydrolysis which was 62.1 ± 0.20 g per 100 g, equating to 29.2 ± 0.09 g per 47 g serving (Table 2).

**Table 2:** Free sugars and oligosaccharides in the developed low-fructose CHO gel.

Sugar/oligosaccharide	Original gel (g/100 g^a^)	Hydrolysed gel (g/100 g^a^)
Glucose	3.84 ± 0.12	8.65 ± 0.34
Sucrose	10.20 ± 0.38	10.70 ± 0.60
Fructose	0.17 ± 0.02	0.15 ± 0.02
Maltose	6.28 ± 0.10	13.3 ± 0.25
Total mono- and disaccharides	20.5 ± 0.57	32.8 ± 0.29
Maltotriose (Mal-3)	6.30 ± 0.08	16.3 ± 0.14
Maltotetraose (Mal-4)	4.49 ± 0.21	13.0 ± 0.33
Maltopentaose (Mal-5)	2.29 ± 0.14	0.00 ± 0.00
**Total maltooligosaccharides**^b^	13.1 ± 0.18	29.4 ± 0.21
**Total** CHO^c^	**n/a**	**62.1 ± 0.20**

a Mean ± SD of three CHO gel samples.

b Maltooligosaccharides are oligosaccharides comprised of 3–10 glucose molecules linked by α 1,4 glycosidic bonds. These totals account only for maltooligosaccharides comprised of 3–5 glucose monomers that are detected by HPAEC-PAD.

c Total CHO calculated from the sum of all sugars and maltooligosaccharides detected by HPAEC-PAD. Any oligosaccharides with a degree of polymerisation >5 glucose monomers are not detected therefore true total CHO content can only be calculated following complete hydrolysis, assumed to be reached after a 120-min incubation at 37 °C with human salivary α-amylase IX.

### 3.6 Feeding tolerance assessment

Euhydration was maintained pre- and post-exercise (P_osmol_: 291 ± 9.3 and 291 ± 7.3 mOsm/kg, respectively). During exercise, 1982 ± 267 mL water was consumed, resulting in exercise-induced body mass loss of 1.3 ± 0.9 %, and a 5.9 ± 4.0 %. decrease in plasma volume. Tympanic temperature increased by 1.4 ± 0.6 °C (*p* < 0.001) post-exercise. Both HR and RPE increased with exercise (*p* < 0.001). Mean distance covered during the 1 h self-paced distance test was 10.3 ± 1.7 km.

**Fig. 4:**
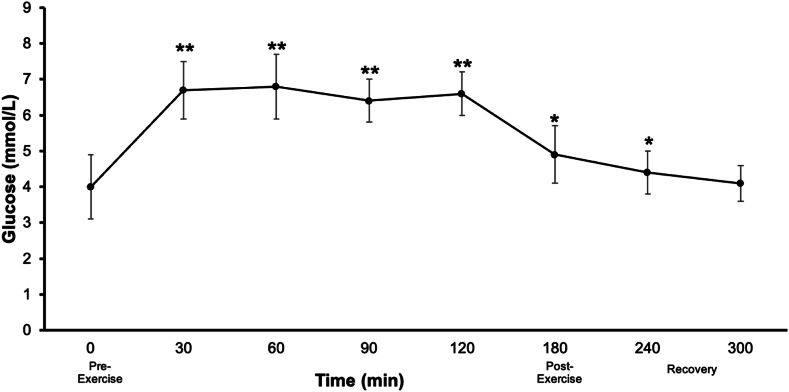
Blood glucose values at pre- (0), during (30, 60, 90, 120), post-exercise (180), and during recovery (240, 300). Mean ± SD (n = 20). **p < 0.001 vs. 0 min, *p* < 0.05 vs. 0 min.

An increase (*p <* 0.001) from the pre-exercise blood glucose value (4.0 ± 0.9 mmol/L) occurred during the first 2 h of running (30-, 60-, 90-, 120-min), with the ingestion of one CHO gel every 20 min (Fig. 4). Post-exercise, blood glucose was 4.9 ± 0.8 mmol/L and gradually decreased during the first and second hour of recovery (4.4 ± 0.6 and 4.1 ± 0.5 mmol/L, respectively).

**Fig. 5:**
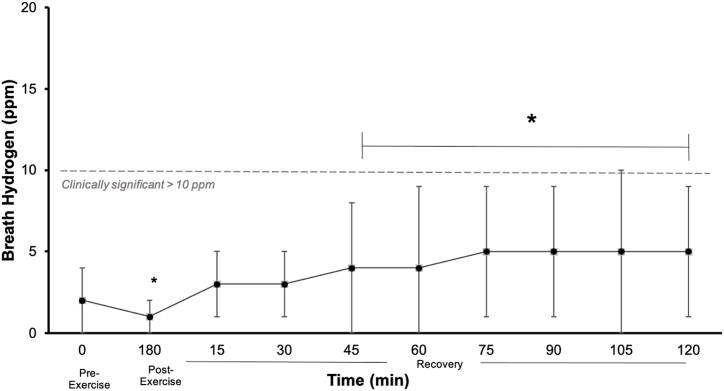
Breath H_2_ in response to 2 h steady state running exercise (60 % *V̇*O_2max_) while consuming 30 g CHO/20 min followed by 1 h running distance test and during 2 h post-exercise recovery. Mean ± SD (n = 20). *p < 0.05 vs. 0 min.

Pre-exercise, the mean breath hydrogen (H_2_) level was 2 ± 2 ppm. Throughout recovery, there was a significant increase (*p* < 0.05) in breath H_2_ at 45-, 60-, 75-, 90-, 105-, and 120-min timepoints compared to baseline, but only 25 % of the cohort demonstrated significant CHO malabsorption (≥10 ppm over baseline) (Fig. 5).

During exercise, 45 % of runners experienced mild taste fatigue, progressively increasing from 60- to 180-min compared to pre-exercise (peak mVAS score: 2 ± 3 at 100- and 120-min vs. 0 at pre-exercise, *p* < 0.05). Tolerance to food and drink decreased from 40- to 120-min of exercise, compared to baseline (*p* < 0.05). Interest in food and drink, appetite, and thirst decreased during exercise, with significance noted for thirst from 40- to 120-min of exercise, compared to pre-exercise (*p* < 0.05) (Appendix A*,* Figure S1). Throughout recovery, all feeding tolerance variables except for taste fatigue were higher than pre-exercise levels (*p* < 0.001) (Table 3).

**Table 3:** Incidence and severity of feeding-tolerance variables in response to 2 h steady-state running (60 % $\dot{V}$O_2max_) with CHO and water ingestion, followed by 1 h self-paced distance test at ambient conditions (23 ± 1 °C T_amb_, 49 % RH).

	Pre-exercise^a^	During exercise^b^	Total Exercise^c^	Total Recovery^c^
**Taste fatigue**	0 (0)	1 (0–8)^d^	8 (0–31)^d^	6 (0–55)^e^
**Interest in food**	1 (0–8)	2 (0–10)	6 (0–30)	54 (0- 90)^e^
**Interest in drink**	2 (0–8)	3 (0–10)	14 (0–51)^d^	37 (1–87)^e^
**Tolerance to food**	6 (0- 10)	6 (0–10)	35 (0–70)^d^	72 (16–90)^e^
**Tolerance to drink**	9 (0–10)	8 (0–10)	44 (0–70)^e^	67 (1–90)^e^
**Appetite**	2 (0–7)	3 (0–8)	9 (0–41)^d^	57 (4–87)^e^
**Thirst**	4 (0–10)	5 (0–10)	15 (0–54)^d^	34 (1–83)^e^

a Pre-exercise: rating scale point score before feeding and exercise.

b During exercise: rating scale point score at 180 min of exercise.

c Total exercise and recovery: overall summative rating scale point score of measured time periods. Data presented as mean and individual reported range (n = 20).

d *p* < 0.05 vs pre-exercise.

e *p* < 0.001 vs. pre-exercise.

High incidence of total- (100 %), upper- (100 %), lower- (90 %), and other-GIS (50 %) was observed but all of mild severity (mVas score: 0–4). Total- and upper-GIS increased from pre-exercise (*p* < 0.05) from 40- to 120-min of exercise (Appendix A, Figure S2). Mild belching (80 %) and upper abdominal bloating (95%) were the most common (mVAS score during exercise: 2 ± 1 and 3 ± 2, respectively). For lower-GIS, lower abdominal bloating (50 %) and urge to defecate (75 %) were of highest incidence but mild (mVAS score during exercise: 1 ± 1 and 1 ± 1, respectively) (Table 4).

**Table 4:** Incidence and severity of gastrointestinal symptoms (GIS) in response to 2 h steady-state running (60 % $\dot{V}$O_2max_) with CHO and water ingestion, followed by 1 h self-paced distance test at ambient conditions (23 °C T_amb_, 49 % RH).

	Incidence During (%)^a^	Pre-exercise^b^	During Exercise^c^	Total Exercise^d^	Total Recovery^d^
**Overall Gut Discomfort**	90	2 (0–8)	2 (0–10)	23 (0–66)^g^	7 (0–44)
**Total GIS**^e^	100	5 (0–31)	8 (0–37)	61 (7–130)	20 (0 - 60
**Total Upper GIS**	100	3 (0–12)	7 (1–18)	40 (5–106)	7 (0–40)
Belching	80	0 (0–2)	1 (0–5)	12 (0–29)^g^	1 (0–10)^e^
Heartburn	35	0 (0–1)	1 (0–4)	2 (0–13)^h^	1 (0–11)
Upper abdominal bloating	95	3 (0–9)	1 (0–7)	19 (0–44)	5 (0–34)
Upper abdominal pain	10	0 (0)	1 (0–7)	1 (0–7)	1 (0–1)
Urge to regurgitate	30	0 (0)	0 (0–0)	2 (0–20)	0 (0)
Regurgitation	20	0 (0)	1 (0–10)	5 (0–60)	0 (0–60)
Projectile vomiting	0	0 (0)	0 (0)	0 (0)	0 (0)
**Total Lower GIS**	90	2 (0–19)	3 (0–3)	17 (0–33)^g^	9 (0–28)^h^
Flatulence	40	0 (0–2)	0 (0)	3 (0–14)^h^	1 (0–4)^h^
Lower abdominal bloating	50	1 (0–9)	1 (0–5)	7 (0–26)	4 (0–19)
Urge to defecate	75	0 (0)	0 (0–0)	5 (0–13)^g^	2 (0–13)^h^
Intestinal pain, right	20	0 (0–5)	1 (0–9)	1 (0–9)	0 (0–5)
Intestinal pain, left	10	0 (0–5)	1 (0–6)	1 (0–6)	0 (0–5)
Defecation, normal stools	20	0 (0)	0 (0)	2 (0–10)	1 (0–10)
Defecation, abnormal stools^f^	10	0 (0)	0 (0)	1 (0–10)	2 (0–10)
**Total Other GIS**	50	0 (0)	2 (0–10)	4 (0–15)^h^	3 (0–24)
Nausea	30	0 (0)	1 (0–4)	1 (0–6)^h^	0 (0–1)
Dizziness	35	0 (0)	1 (0–8)	2 (0–9)^h^	3 (0–23)
Stitch	25	0 (0)	1 (0–10)	2 (0–14)^h^	0 (0)

a Incidence during: Total number (%) of participants reporting ≥1 on the modified visual analogue scale (mVAS) during exercise for any GIS type during running.

b Pre-exercise: mVAS rating scale point score before feeding and exercise (0 min).^c^ During exercise: mVAS rating scale point score in response to 2 h steady-state running (60 % $\dot{V}$O_2_max) while ingesting 90 g/h carbohydrate and water (10 % w/v), followed by 1 h self-paced distance test with *ad libitum* water (180 min).

c During exercise: mVAS rating scale point score in response to 2 h steady-state running (60% *V̇*O_2_max) while ingesting 90 g/h CHO and water (10% w/v), followed by 1 h self-paced distance test with ad libitum water (180 min)

d Total exercise and recovery: overall summative mVAS rating scale point score of measured time periods and individual reported range.

e Summative accumulation of upper-, lower- and other-GIS.

f Summative accumulation of abnormal stools referring to loose watery stools, diarrhea and/or fecal blood loss.

g *p* < 0.001 vs. pre-exercise.

h *p* < 0.05 vs pre-exercise.

## 4 Discussion

Despite the diverse array of commercially-available CHO gels, some individuals experiment with creating their own CHO supplement to cater to specific needs. As such, a structured process to ensure safety, quality, and accurate reproducibility is warranted. In this applied experimental study, we established a systematic CHO gel development procedure and investigated tolerance to the formulated product. The low-fructose CHO gel was well-tolerated and promoted blood glucose availability, without clinically-significant CHO malabsorption or severe GIS during exercise.

Along with recent revisions in guidelines [[1], [5]], insights on the impact of fructose and other FODMAPs on gastrointestinal physiology and symptoms highlight the need for increased variability in CHO blends in sports nutrition products. We found only 46 % of CHO gels in the local commercial market meet the minimum recommended hourly CHO intake during prolonged exercise (30 g/h) per serve and/or based on serving suggestion, and did not contain fructose (per ingredient list). Similar to previous findings [27], there were vast differences in ingredients, nutrition, CHO type, dose, and flavour. These differences may impact the fate of a product from ingestion (e.g., sensorial qualities and feeding tolerance), digestion and absorption (i.e., gastric emptying, intestinal transit, CHO absorption, and bacterial fermentation), skeletal muscle oxidation, and ultimately, exercise performance. A previous study on CHO gels commercially available in the UK also saw variety among products with only 3 out of the 23 (13 %) CHO gels not containing fructose based on ingredient list [27]. This highlights country-specific differences and the need to conduct a survey of commercially-available CHO gels in each location prior to developing an individualized formulation. The current findings however are limited by the nutrition and ingredient information on product labels and further validation through testing can be explored.

Easily-digestible CHOs are preferred during exercise to optimise fuel availability and minimize GIS risk [1,5]. In this study, maltodextrin was chosen as the primary ingredient of the CHO gel due to its low taste intensity [28] compared to other CHO sources. This reduces the degree of sweetness of the formulation, which is an important factor in preventing taste fatigue when the product is to be consumed repeatedly over a long duration. Furthermore, maltodextrin easily dissolves in water and forms a viscous mixture [29], which aids in the desired consistency of the end product. It is also rapidly digested and absorbed [30], helping promote greater fuel availability during exercise. The dextrose equivalent (DE) value of maltodextrin may impact physicochemical and sensory qualities of the end product [29,31] and thus, needs to be considered when sourcing ingredients. As an example, maltodextrins with a lower DE would be less sweet and viscous, compared to a greater DE (i.e., 20). The maltodextrin used in our formulation has a DE value of 18–20, which equates to a degree of polymerisation (DP) of ∼6, while glucose has a DE of ≥20. Further studies could also investigate adjusting the formulation to increase the calorie to serving size ratio.

Sensory evaluation helps assess product quality and acceptance. Sample B (24 % *w/v*) was selected as the final formulation due to its favourable attributes and overall had the least volume per serving, making it more portable for exercise. Assessing parameters related to the packaging of the product and its feasibility of use within exercise, as well as identifying more specific panellists within the targeted consumer group (i.e., endurance athletes) to test out the actual product during exercise, is suggested for future studies.

The physicochemical and microbiological properties of food play a crucial role in its biological impact, influencing digestion, absorption, and safety. The product as consumed has a low osmolality (171 ± 2 mOsm/kg), minimizing the risk of delayed gastric emptying and upper-GIS linked to high nutrient density and/or osmolality of food and/or fluids [24,32]. Many commercially-available CHO gels have high osmolality values (>2000 mOsm/kg) [27], and if not consumed with adequate fluids, this increases GIS risk [24,33]. The product's viscosity (5466 ± 34 mPa·s) promotes ease of ingestion during exercise, reducing spillage, and enhancing contact time with CHO-sensing receptors in the mouth. In terms of food safety, the product's pH and a_w_ made it non-fertile growth media for food-associated pathogens [34]. While the ‘kill step’ was likely sufficient to result in very low microbial counts, validation of the time-temperature conditions is recommended in the future by exposing the product to outdoor conditions similar to when it will be used by athletes.

Various analyses for nutrition labelling and quality assurance are essential in product development. This study's strength lies in validating the product's actual CHO content against ingredient labels. *In vitro* hydrolysis with human salivary *α*-amylase ensured all maltodextrins, and other oligo- and polysaccharides, were accounted for, and sugars were detected by HPAEC-PAD. Results revealed minimal fructose (0.17 % *w/w*) and 29.2 g of total CHOs per 47 g serving. No isomaltose was detected following the *in vitro* hydrolysis, suggesting very few or no branched dextrins were present in the gel. Maltooligosaccharides consist of 3–10 glucose molecules linked by *α*-1,4 glycosidic bonds (i.e., unbranched maltodextrins) [21]. Since the formulation contained maltodextrin with a DE of 18–20 and glucose syrup with a DE of ≥20, most maltooligosaccharides in the gel will have a DP of ≤6 glucose monomers. The complete hydrolysis of Mal-5, and cessation in glucose and maltose production after 120 min of incubation with *α*-amylase, demonstrates that all maltooligosaccharides were quantified. The CHO content of the gel, in the form in which it would be ingested, comprised 33.0 % sugars (monosaccharides and disaccharides), 21.0 % maltooligosaccharides with a DP ≤ 5, and 46.0 % maltooligosaccharides with a DP > 5 glucose units. Stoichiometrically, a serving of the CHO gel would provide 28.1 g of glucose and 2.71 g of fructose (mostly from sucrose), assuming 100 % digestion and absorption of all CHO upon ingestion.

The sugar (29.7 g/100 g) and total CHO (63.8 g/100 g) contents, based on product labels slightly differ from the lab quantification findings. The undigested gel contains 20.5 g/100 g available sugars (monosaccharides and disaccharides). It is difficult to compare these values because of the varying definitions of sugar and quantification techniques. After hydrolysis, the sugar content increased to 32.8 g/100g, and total CHO in the gel is 62.1 g/100 g (∼97 % of the anticipated value). This slight undervalue is likely accounted for by the presence of some small peaks in the SA10 chromatograms for which maltooligosaccharide standards were not available. Irregularities may stem from presence of some trace undetected branched maltodextrins, incomplete hydrolysis of larger maltodextrins, presence of undigested CHOs from thickening agents in the raw ingredients, and/or impurities in the sugar standards. CHO quantification results confirm that the product qualifies as a formulated sports food and a high CHO supplement [35,36]. Validating nutrition content through testing to ensure formulation quality is suggested as best practice.

Maintaining blood glucose levels during endurance exercise is crucial for performance. Prolonged efforts rely on the oxidative metabolism of both fat (intramuscular triglycerides and adipose tissue stores) and CHO (muscle glycogen, blood glucose from liver, and exogenous CHO) to provide ATP to working skeletal muscles [37]. In endurance exercise, skeletal muscle uptake exceeds glucose provisions from breakdown of skeletal muscle and liver glycogen, resulting in hypoglycaemia, and decreased physical performance [37]. Adequate CHO ingestion can mitigate this impairment. We showed that ingestion of the developed product (90 g/h) increased blood glucose values from pre-exercise levels during the run (65 %) and post-exercise (23 %), and remained elevated during the first hour of recovery (10 %). This is comparable to previous studies with a similar exercise and supplementation protocol, but using a 2:1 glucose to fructose supplement [3,10]. Multiple transportable CHOs aim to increase CHO absorption and availability [8] but are also linked with feeding intolerance, CHO malabsorption, and GIS [3,6,10]. From a research perspective, looking at other gastrointestinal-related functional measures and skeletal muscle metabolism outcomes is suggested to fully understand the effect of supplementing with a low-fructose CHO gel.

Breath H_2_ testing is used to assess CHO malabsorption, with a level ≥10 ppm considered clinically significant and linked to GIS [26]. Breath H_2_ significantly increased during recovery (cohort mean: 5 ppm peak H_2_), but only 25 % exhibited clinically significant CHO malabsorption (mean peak breath H_2_: 14 ± 3 ppm). This may be attributed to oversaturation of intestinal CHO transporters (e.g., SGLT1 and/or GLUT2), particularly during exercise [38,39]. The low-fructose formulation likely contributed to the low incidence of CHO malabsorption compared to previous studies using a 2:1 glucose-fructose formulation (68 % and 61 %, respectively) [3,10]. Fructose, when not completely absorbed, increases osmotic load and changes gastrointestinal motility, especially in individuals with functional gastrointestinal disorders, compared to asymptomatic healthy individuals [12]. The variability in the breath H_2_ response underscores the importance of personalising CHO supplementation based on individual tolerance [6]. To comprehensively understand tolerance to a CHO gel at a gastrointestinal level, interpreting breath H_2_ data alongside GIS outcomes and other functional measures (e.g., perturbations in intestinal epithelial integrity, gastric emptying, and orocecal transit time) is recommended.

Feeding tolerance is crucial for successful fuelling during endurance and ultra-endurance events (>2 h) [5]. Taste fatigue affected 45 % of the runners, but only 3 out of 20 found it severe (>4 mVAS rating scale point). Tolerance to food (mean summative accumulation score: 35) and to drink (mean summative accumulation score: 44) decreased from baseline, yet not to the extreme low whereby participants could no longer drink or eat further. Interest in food/drink and appetite were not significantly impacted, suggesting tolerance to the CHO gel but not to the amount of water provided. GIS were present in 90 % of runners but were mainly mild and unlikely to affect feeding tolerance. The most common upper-GIS during exercise (belching and heartburn) are likely due to a large gastric load, increasing intragastric pressure and/or intestinal load, which can hamper gastric emptying and orocecal transit time [40,41]. This was expected given the high fluid intake (870 mL/h) [42,43] provided with the gels as part of the feeding-challenge protocol, which can induce artefact GIS. The authors speculate that the mild GIS observed in the cohort can be linked to the discrepancy in volume of intake between the study protocol and the self-reported mean CHO and fluid intakes of the athletes during exercise which were 37 ± 19 g/h of CHO and 594 ± 217 mL/h of fluids. Furthermore, flatulence and urge to defecate increased during exercise, potentially related to malabsorption and water being osmotically-driven into the bowel, alongside bacterial fermentation of CHOs producing gas [44]. Monitoring GIS in various exercise settings (e.g., intensity, temperature, and/or altitude) is recommended for individualised assessment of tolerance to a CHO supplement.

In line with published guidelines for endurance and ultra-endurance sports [1,5], the authors acknowledge that individualised fuelling strategies are vital for an athlete; specifically, identifying individual tolerance levels in terms of amount and composition. The developed CHO gel's fructose content even at a higher hourly intake rate (>90 g/h during exercise) is still considered low in comparison to fructose loads that have been shown to induce clinically-relevant symptoms (e.g., 0.5 g/100 g naturally occurring free fructose in excess of glucose, >3 g fructose load in an average serving quantity of the food or beverage, 0.5 g of fructans per serving) [18]. Furthermore, the data in the current study shows that only 25 % of the cohort demonstrated significant CHO malabsorption (≥10 ppm above baseline). This is considered low compared to previous gut-challenge studies using a CHO supplement with a 2:1 glucose to fructose ratio by Costa et al. [3] and Miall et al. [10] wherein CHO malabsorption rates were 68 % and 61 %, respectively. In both studies, a greater percentage of the cohort also reported occurrence of moderate and severe GIS. Based on this, the authors speculate that chronic use of the developed gel and any other gel, above tolerance levels of an individual would result in GIS, but likely of more severity if the gel contains high fructose loads.

In summary, our study demonstrated that readily-available ingredients can be used to create a low-fructose CHO gel with good sensory properties and positive gastrointestinal-, feeding tolerance-, and fuel availability-related outcomes during exercise. Although clinically-significant CHO malabsorption was observed in some runners, only mild GIS occurred during exercise. We acknowledge that the feeding tolerance testing was conducted in a laboratory setting and outcomes may differ in actual race conditions and/or long training sessions, due to other factors, as previously described [22]. To further improve the sports nutrition product development process, a comprehensive assessment that considers the various limiting factors to fuel availability should be conducted, incorporating factors like intake behaviour and tolerance, gastrointestinal transit, and circulatory availability [6]. In addition, future work should include a comparator trial using a placebo and/or a CHO gel with a different blend as part of the developmental process to further validate findings. Industry product developers may also benefit from integrating these processes for product renovation or innovation and making evidence-based product claims.

## 5 Conclusion

We developed and tested a low-fructose CHO gel among endurance athletes with findings indicating that the product was well-accepted in terms of its sensory properties and nutritionally comparable to commercially-available CHO supplements. Quantification of sugars confirmed the low level of fructose and a total CHO content of 29.2 g per 47 g serving. Most runners showed no CHO malabsorption and severe GIS during a feeding-challenge protocol, involving the ingestion of the low-fructose CHO gel every 20 min (90 g/h) during 2 h steady-state running. As taste fatigue was a concern, optimisation of ingredients and flavour is recommended for future product reformulation.

## Funding

This research did not receive any specific grant from funding agencies in the public, commercial, or not-for-profit sectors. IGM is supported by the Monash International Tuition Scholarship and Monash Graduate Scholarship. JRB is supported by a National Health and Medical Research Council Emerging Leadership Fellowship (APP2025943).

## Data availability

The data that support the findings of this study are available on request from the corresponding author, RC. The data are not publicly available due to containing information that could compromise the privacy of research participants.

## Ethics statement

Ethical approval for the involvement of human subjects in this study was granted by Monash University Human Research Ethics Committee (MUHREC), reference number 35216, 08/19/2022.

## CRediT authorship contribution statement

**Isabel G. Martinez:** Writing – review & editing, Writing – original draft, Project administration, Methodology, Investigation, Formal analysis, Data curation, Conceptualization. **Michael J. Houghton:** Writing – review & editing, Writing – original draft, Resources, Methodology, Investigation, Formal analysis, Data curation. **Matteo Forte:** Writing – review & editing, Investigation, Formal analysis, Data curation. **Gary Williamson:** Writing – review & editing, Supervision, Resources, Methodology, Funding acquisition, Formal analysis. **Jessica R. Biesiekierski:** Writing – review & editing, Writing – original draft, Supervision, Resources, Methodology, Investigation, Funding acquisition, Conceptualization. **Ricardo J.S. Costa:** Writing – review & editing, Writing – original draft, Supervision, Funding acquisition.

## Declaration of competing interest

This research did not receive any specific grant from funding agencies in the public, commercial, or not-for-profit sectors. In regards to the current manuscript submission, all authors have no conflicts of interest to declare.
