# Supplementary material for: Development of a low-fructose carbohydrate gel for exercise application
Source: Heliyon. 2024 Jun 22;10(13):e33497. doi: 10.1016/j.heliyon.2024.e33497 (PMC11260965; doi:10.1016/j.heliyon.2024.e33497)
Supplement: Multimedia component 1 [file mmc1.pdf]

Monash University Human Research Ethics Committee

Approval Certificate

This is to certify that the project below was considered by the Monash University Human Research Ethics Committee. The Committee was satisfied that the proposal meets the requirements of the *National Statement on Ethical Conduct in Human Research* and has granted approval.

**Project ID:**	35216
**Application Type:**	HREC Review
**Project Title:**	Gut Training- Impact of macronutrient composition and density
**Chief Investigator:**	Assoc Professor Ricardo Da Costa
**Approval Date:**	19/08/2022
**Expiry Date:**	19/08/2027

Terms of approval - failure to comply with the terms below is in breach of your approval and the *Australian Code for the Responsible Conduct of Research.*

1. The Chief Investigator is responsible for ensuring that permission letters are obtained, if relevant, before any data collection can occur at the specified organisation.
2. Approval is only valid whilst you hold a position at Monash University.
3. It is responsibility of the Chief Investigator to ensure that all investigators are aware of the terms of approval and to ensure the project is conducted as approved by MUHREC.
4. You should notify MUHREC immediately of any serious or unexpected adverse effects on participants or unforeseen events affecting the ethical acceptability of the project.
5. The Explanatory Statement must be on Monash letterhead and the Monash University complaints clause must include your project number.
6. Amendments to approved projects including changes to personnel must not commence without written approval from MUHREC.
7. Annual Report - continued approval of this project is dependent on the submission of an Annual Report.
8. Final Report - should be provided at the conclusion of the project. MUHREC should be notified if the project is discontinued before the expected completion date.
9. Monitoring - project may be subject to an audit or any other form of monitoring by MUHREC at any time.
10. Retention and storage of data - The Chief Investigator is responsible for the storage and retention of the original data pertaining to the project for a minimum period of five years.

Kind Regards,

Professor Nip Thomson

Chair, MUHREC

CC: Ms Isabel Martinez, Dr Jessica Biesiekierski

List of approved documents:

Document Type	File Name	Date	Version
Supporting Documentation	Gut training - Recruitment Poster	28/07/2022	1
Explanatory Statement	Gut training - Explanatory statement	28/07/2022	1
Consent Form	Gut Training - Consent form	28/07/2022	1
Questionnaires / Surveys	2 mVAS GIS tool	28/07/2022	1
Questionnaires / Surveys	1 GTS - Intervention compliance and food diary	28/07/2022	1
Questionnaires / Surveys	1 GTS - Pre-exercise questionnaire	28/07/2022	1
Questionnaires / Surveys	1 GTS - Screening questionnaire	28/07/2022	1
Questionnaires / Surveys	0 GTS 2.1 Study Design	28/07/2022	1
Supporting Documentation	Data collection sheet 1	28/07/2022	1
Supporting Documentation	Data collection sheet 2	28/07/2022	1

Supporting Documentation	SOP - Capillary blood	28/07/2022	1
Supporting Documentation	SOP - Treadmill	28/07/2022	1
Supporting Documentation	SOP - Weight measurement	28/07/2022	1
Supporting Documentation	SOP - Height	28/07/2022	1
Supporting Documentation	SOP - Hydration status	28/07/2022	1
Supporting Documentation	SOP - Breath samples analyser	28/07/2022	1
Supporting Documentation	SOP - VO2max test	28/07/2022	1
Supporting Documentation	SOP - Indirect calorimetry	28/07/2022	1
Supporting Documentation	SOP - Venipuncture	28/07/2022	1
Supporting Documentation	SOP - Capillary Blood sampling	28/07/2022	1
Supporting Documentation	RA18903 SCS Use of exercise equipment, including treadmill and exercise bike	28/07/2022	1
Supporting Documentation	RA 39910 - Overall study risk assessment	28/07/2022	1
Explanatory Statement	0 GTS - Explanatory statement_v2	10/08/2022	2
Consent Form	Gut Training - Consent form v2	10/08/2022	2
Supporting Documentation	Gut training - recruitment poster v2	10/08/2022	2
