# Supplementary material for: Development of a low-fructose carbohydrate gel for exercise application
Source: Heliyon. 2024 Jun 22;10(13):e33497. doi: 10.1016/j.heliyon.2024.e33497 (PMC11260965; doi:10.1016/j.heliyon.2024.e33497)
Supplement: Multimedia component 2 [file mmc2.docx]

**Development of a low-fructose carbohydrate gel for exercise application**

Isabel G. Martinez^1^, Michael J. Houghton^1,2^, Matteo Forte^3^, Gary Williamson^1,2^, Jessica R. Biesiekierski^1^, Ricardo J.S. Costa^1^

^1^ Department of Nutrition, Dietetics and Food, Monash University, Level 1, 264 Ferntree Gully Road, Notting Hill, VIC 3168, Australia

^2^ Victorian Heart Institute, Monash University, Victorian Heart Hospital, 631 Blackburn Road, Clayton, VIC 3168, Australia

^3^ Department of Land, Environment, Agriculture and Forestry, Università degli Studi di Padova, Viale dell’Università 16, 35020, Legnaro, PD, Italy

**Corresponding author:**

Ricardo J.S. Costa: Monash University, Department of Nutrition & Dietetics, Level 1, 264 Ferntree Gully Road, Notting Hill, 3168, Victoria, Australia. Telephone: 00 61 3 99024270. Email: [ricardo.costa@monash.edu](mailto:ricardo.costa@monash.edu)

**Supplementary Information.**

Table S1. Nutrition information of the developed low-fructose carbohydrate gel

| **NUTRITION INFORMATION** | | | | |
| --- | --- | --- | --- | --- |
| Servings per package: | 1 |  | | |
| Serving size: | 47 g |  | | |
|  | Average Quantity  per Serving | | Average Quantity  per 100 g | |
| Energy | 516 | kJ (123 Cal) | 880 | kJ (211Cal) |
| Protein | 0.0 | g | 0.0 | g |
| Fat, total | 0.0 | g | 0.0 | g |
| - saturated | 0.0 | g | 0.0 | g |
| Carbohydrate | 30.0 | g | 63.8 | g |
| - sugars | 10.0 | g | 29.7 | g |
| Dietary Fibre | 0.0 | g | 0.0 | g |
| Sodium | 19 | mg | 40 | mg |

Table S2. Physicochemical and Microbiological Properties of the Carbohydrate Supplement

| **Test** | **Result^a^** |
| --- | --- |
| Osmolality (mOsm/kg) | 171 ± 1.5 |
| Viscosity (mPa·s) | 5466 ± 34 |
| pH | 4 ± 0 |
| Water activity (a_w_) | 0.90 |
| Aerobic Plate Count (cfu/g) | <10 |
| Yeast and Mould (cfu/g) | <10 |
| Coliform (cfu/g) | <10 |
| E coli (cfu/g) | <10 |
| Coagulase Positive Staph (cfu/g) | <100 |
| Salmonella spp. (/25g) | Not detected |

^a^ Mean ± SD of three carbohydrate gel samples.

mOsm = milliosmole, mPa·s = millipascal-seconds, Cfu = colony forming unit.

**Figure legends.**

**Figure S1.** Feeding tolerance variables, including taste fatigue (A), interest in food (B), interest in drink (C), tolerance to food (D), tolerance to drink (E), appetite (F) and thirst (G) ranked on a 10-point Likert-type rating scale (mVAS), during 2 h steady-state running (60% V̇O2max) with carbohydrate (90 g/h) and water (10% w/v) ingestion, followed by 1 h self-paced distance test with ad libitum water in ambient conditions (23°C T_amb_, 49% RH).

Mean ± SD (n = 20). * p< 0.05 vs. pre-exercise (0 min).

***Figure S2***. Overall gut discomfort (A), total (B), upper (C), and lower (D) gastrointestinal symptom severity ranked on an exercise-specific mVAS (Gaskell et al., 2019), during 2 h steady-state running (60% V̇O2max) with carbohydrate (90 g/h) and water (10% w/v) ingestion, followed by 1 h self-paced distance test with *ad libitum* water in ambient conditions (23°C T_amb_, 49% RH).

Mean ± SD (n = 20). * p< 0.05 vs. pre-exercise (0 min).

**Figure S1.**


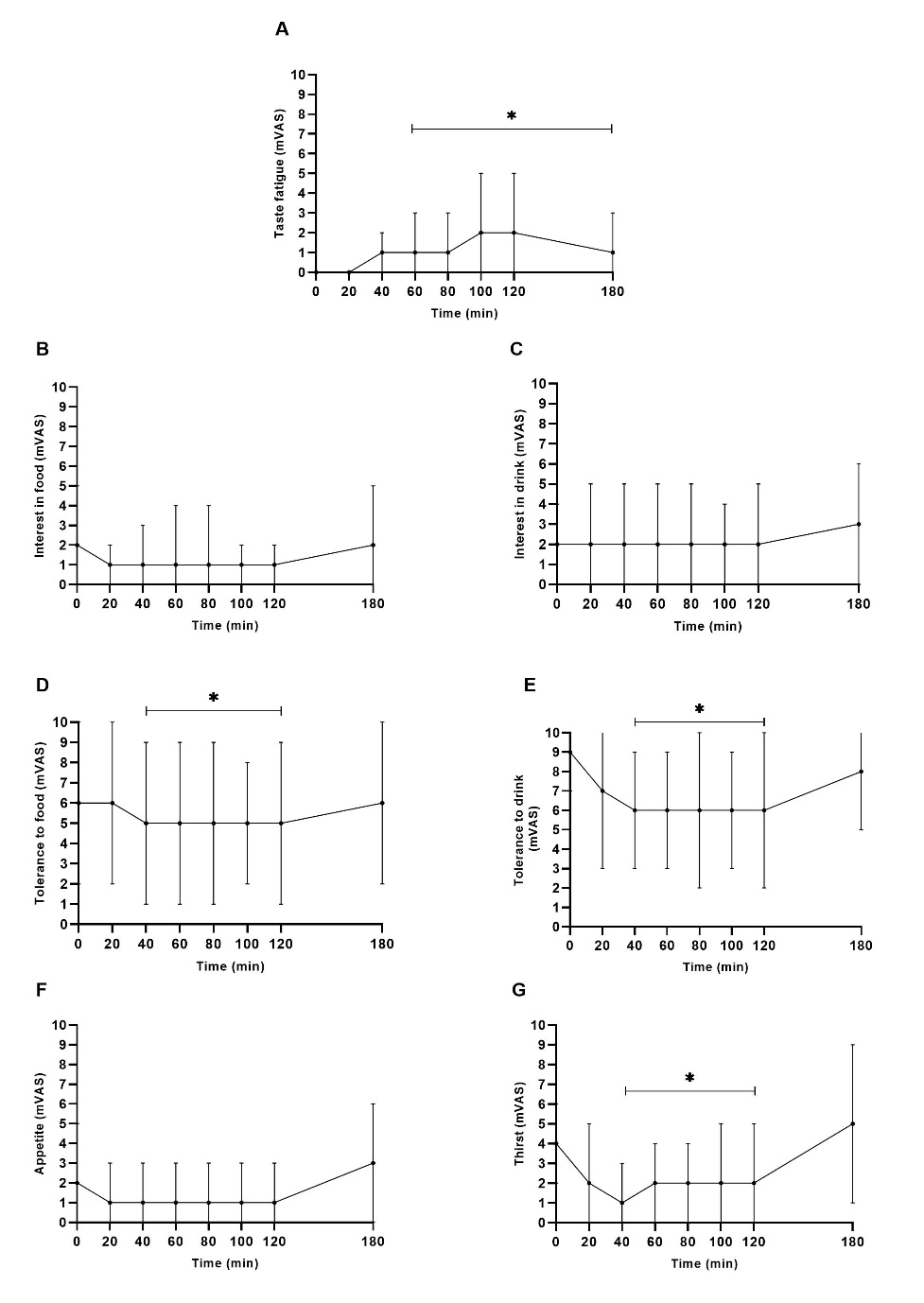


**Figure S2.
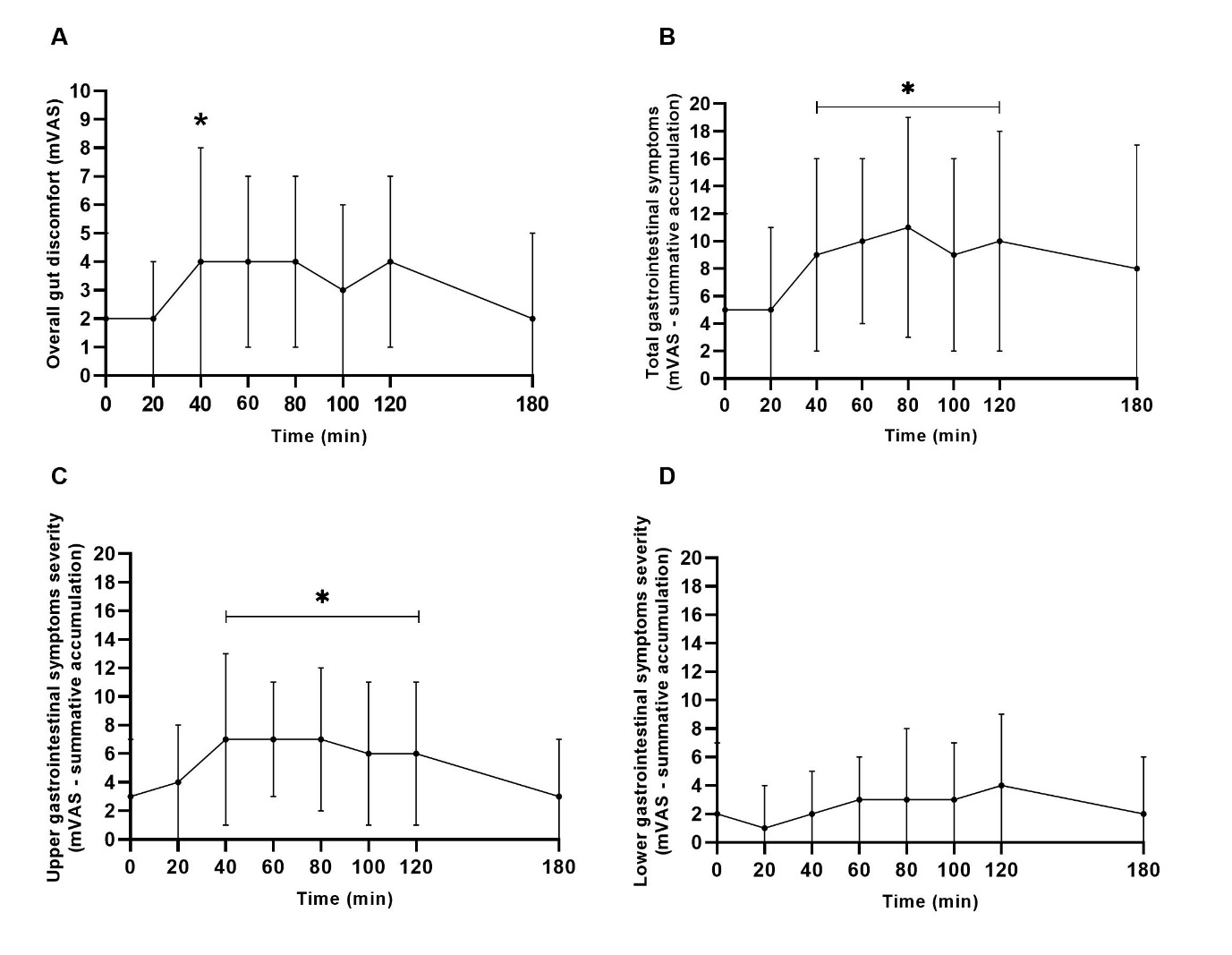
**
